# The Cancer Prevention, Anti-Inflammatory and Anti-Oxidation of Bioactive Phytochemicals Targeting the TLR4 Signaling Pathway

**DOI:** 10.3390/ijms19092729

**Published:** 2018-09-12

**Authors:** Chung-Yi Chen, Chiu-Li Kao, Chi-Ming Liu

**Affiliations:** 1 School of Medical and Health Sciences, Fooyin University, Ta-Liao District, Kaohsiung 83102, Taiwan; xx377@fy.edu.tw; 2 Department of Nursing, Tzu Hui Institute of Technology, Pingtung County 92641, Taiwan; kkjj77677@gmail.com; 3 School of Medicine, Yichun University, Yuanzhou District, Yichun 336000, China

**Keywords:** toll-like receptor, chemoprevention, anti-inflammatory

## Abstract

Toll-like receptors (TLRs) are a well-known family of pattern recognition receptors that play an important role in a host immune system. TLR triggering leads to the induction of pro-inflammatory cytokines and chemokines, driving the activation of both innate and adaptive immunity. Recently, an increasing number studies have shown the link between TLRs and cancer. Among them, the toll-like receptor 4 (TLR4) signaling pathway is associated with inflammatory response and cancer progression. Dietary phytochemicals are potential modulators of immunological status with various pharmacological properties including anti-cancer, anti-oxidant and anti-inflammatory. Curcumin, 6-gingerol, 6-shogaol, 1-dehydro-10-gingerdione, epigallocatechin gallate (EGCG), luteolin, quercetin, resveratrol, caffeic acid phenethyl ester, xanthohumol, genistein, berberine, and sulforaphane can inhibit TLR4 activation. The aim of the present review is to describe the role of the TLR4 signaling pathway between inflammatory response and cancer progression. We further introduce bioactive phytochemicals with potential anti-inflammation and chemoprevention by inhibiting TLR activation.

## 1. Introduction

The immune system defends our body against pathogens, such as viruses, bacteria, and fungus. Monocytes, macrophages, dendritic cells (DCs), neutrophils, and natural killer (NK) cells play an important role in maintaining the innate immune system [1,2,3,4,5]. These immune cells can recognize and respond to invasion, providing efficient protection by specific receptors such as Toll-like receptors (TLRs) [6]. TLRs were named after the toll receptor of Drosophila. The Toll-like protein was shown to induce pro-inflammatory gene expression after ligation with specific pathogen-associated molecular patterns (PAMPs) [7,8]. TLRs are not only expressed in immune cells but also in epithelial cells. Tumor progression involves complex interactions between tumor cells, immune cells, and the tumor microenvironment. TLRs may also cause the development of cancers and inflammatory diseases [9,10,11]. TLRs play anti-tumor or tumor promoter roles in different cancer cells [12].

Several studies have reported that many phytochemicals have anti-inflammatory, anti-oxidation, chemopreventive and anti-cancer properties [13,14]. Recently, some phytochemicals can inhibit pattern recognition receptor (PRR) activation by targeting the receptor or the downstream signaling molecules [15]. In this review, we will discuss the different functions of TLR4 in cancer progression and inflammation. Furthermore, we also summarize and discusses the recent findings of curcumin, 6-gingerol, 6-shogaol, 1-dehydro-10-gingerdione, EGCG, luteolin, quercetin, resveratrol, caffeic acid phenethyl ester, xanthohumol, genistein, berberine, and sulforaphane in inhibiting the activation of TLR4.

## 2. Inflammation and Cancer Progression

Physical, chemical, and infectious agents can prompt inflammation. Lipopolysaccharides (LPS) are found in the cell wall of Gram-negative bacteria and can interact with the receptor of immune cells [16]. When inflammation occurs, inflammatory cytokines will be released, including TNF-α, IL-6, and IL-1 from the immune cells [17]. Inflammation can be chronic or acute depending on the characteristics of response and stimulation. Signs of acute inflammation are heat, redness, swelling and pain. Bradykinin, prostaglandins, thromboxanes, and leukotrienes are also involved in the acute inflammatory response. Arachidonic acid is released from the membrane by phospholipase A2. The released arachidonic acid is used as a substrate by the cyclooxygenase (COX) [18]. The prostanoids produced from arachidonic acid cause inflammatory and pain [18].

If the infection/stimulation is not completely cleared by the acute response or it persists for a long time, a chronic inflammation may result. It is believed that inflammation can lead to carcinogenesis [19,20,21]. Inflammation is known to contribute to carcinogenesis by generation of reactive oxygen species (ROS) that can damage DNA, genomic aberrations, and carcinogenesis. It is now established that chronic infection by bacterial or virus infection cause increasing cancer risk. For example, chronic *Helicobacter pylori* infection is associated with gastric cancer and mucosa-associated lymphoid tissue lymphoma (MALT) [22,23,24]. Chronic hepatitis B virus (HBV) infection increase the risk of hepatocellular carcinoma [25,26]. Tumor-promotion is associated with production of cytokines by immune/inflammatory cells that activate transcription factors, such as NF-κB (nuclear factor kappa B), STAT3 (Signal Transducer and Activator of Transcription 3), and AP-1 (activator protein 1). These transcription factors will upregulate the COX-2/PGE2 (prostaglandin E2) signal pathway in inflammation and cancer cells [27,28]. Inhibition of transcription factors and inflammatory cytokine expression can decrease tumor development and progression. Nonsteroidal anti-inflammatory drugs (NSAIDs) are investigated for the prevention of cancer progression and metastasis particularly in the case of colon cancer. However, long-term NSAIDs treatment develops peptic ulcers [29,30]. Many phytochemicals with anti-inflammatory effects are further examined for their anti-cancer properties.

## 3. Toll-Like Receptor

TLRs are type І protein, PRRs and they can detect pathogens by recognizing PAMPs or damage-associated molecular patterns (DAMPs) [31,32,33]. They can further activate innate immune responses for host defense [34,35,36]. TLRs are a family of transmembrane receptors and 13 TLRs have been identified in humans. TLRs contain extracellular leucine-rich repeat (LRR) motifs and cytoplasmic Toll/interleukin-1 receptor (TIR) homology domain. TLRs are well expressed in different cells or tissues such as dendritic cells, macrophages, natural killing, and epithelium cells [37,38,39]. After activating the TLRs in these cells, they can produce cytokines or pro-inflammatory mediators (Table 1). There are two types of TLRs based on the location of the cell. TLR1, TLR2, TLR4, TLR5, and TLR6 are located on the cell surface but TLR3, TLR7, TLR8, and TLR9 are within endosomes [40]. Furthermore, TLR2 recognizes bacterial lipoproteins, TLR4 recognizes lipopolysaccharides (LPS), and TLR9 recognizes CpG-containing DNA (CpG-ODN) [41,42].

Macrophages, B cells, mast cells, NK cells, monocytes, neutrophils, and basophils express TLRs. Monocytes strongly express TLR2. Neutrophils express all TLRs except TLR3. NK cells strongly express TLR1 [12]. It is well known that some diseases are associated with TLRs activation, such as neuroinflammation, cardiovascular diseases and cancers [43,44,45].

### 3.1. TLR4 Signaling Pathways

**Figure 1:**
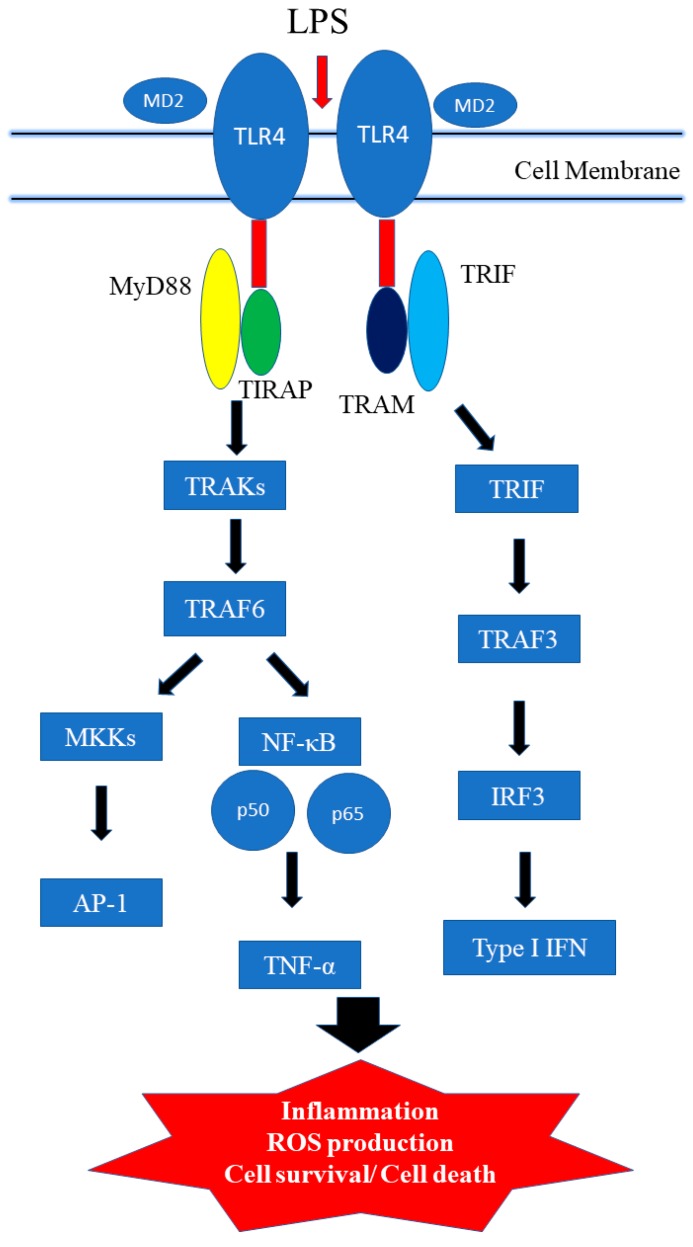
Toll-like receptor (TLR) 4 signaling pathway. LPS binding to MD-2 promotes dimerization of TLR4/MD-2 (red arrow) and actives MyD88 (MyD88-dependent pathway) or TRIF (MyD88-independent pathway) signaling pathways (black arrow).

Toll-like receptor signaling pathways play an important role in innate immune cells. TLR4 activation can induce one or more of four adaptor proteins: myeloid differentiation factor 88 (MyD88), TICAM1 (also known as TIR domain-containing adapter molecule 1), TIRAP (TIR domain-containing adaptor protein), and TICAM2 (TIR domain-containing adapter molecule 2). Lipopolysaccharide (LPS), bacterial endotoxin, can stimulate immune response [46,47,48,49]. The common pathway is that LPS binds to LPS binding protein (LBP) known as differentiation 14 (CD14). CD14 can enhance TLR4 signaling. Furthermore, the co-receptor myeloid differentiation factor-2 (MD-2) promotes the translocation of TLR4 [50]. LPS recognizes the hetertrimer of CD14/TLR4/MD-2. LPS is recognized by Toll-like receptors on the cell surface, which leads to two distinct signal pathways; the myeloid differentiation primary response 88 (MyD88) pathway, and the Toll/IL-1R domain—containing adapter-inducing IFN-β (TRIF) pathway (Figure 1) [46,48].

MyD88 activates IL-1 receptor-associated kinase (IRAK) and it further interacts with tumor necrosis factor receptor-associated factor 6 (TRAF6), resulting in the activation and translocation of NF-κB. IRAK-1, IRAK-2, IRAK-3 and IRAK-4 belong to IRAK family. IRAK-1 and IRAK-4 stimulate TRAF6. Though MyD88 dependent signal pathway, it activates NF-κB and cytokine production [51]. NF-κB is a transcription factor that regulates genes responsible for immune response. NF-κB will activate inducible nitric oxidase synase (iNOS), cyclooxygenase 2 (COX-2), IL-6, and TNF-α. On the other hand, TRAF6 can activate Mitogen-activated protein kinase (MAPKs), p38 and extracellular signal regulated kinase (ERK) signal pathway [52].

Alternately, MyD88-independent pathway (TRIF-dependent pathway) also be observed after TLR4 activation. LPS activates tank-binding kinase-1 (TBK1) and I-kappa-B kinase epsilon (IKKε). Furthermore, it activates interferon regulatory transcription factor 3 (IRF3) and IRF7. IRF3 and IRF7 will activate the transcription of interferon-α (IFN-α) and interferon-β (IFN-β) production. MyD88-dependent and MyD88-independent pathways also contribute to host defense and involve the immune response [53].

### 3.2. TLR4, NF-κB in Inflammatory Process and Tumorigenesis

The immune system can control tumor progression and growth. Chronic inflammation and infection can contribute to the development of tumors. TLRs are potent activators of inflammatory response. TLRs activation can result in the production of cytokines, chemokines and interferons and transcription factor NF-κB. NF-κB pathways plays an important role in various diseases by regulation of immunity, proliferation, differentiation, and apoptosis. The NF-κB family consists of five important members including c-Rel, p50, p52, p65/RelA, and RelA. NF-κB is a transcription factor that binds to DNA and activates gene transcription. NF-κB is bound to I kappa B (IκB) in the cytoplasm without stimulation. A variety of stimulation such as ROS and inflammatory cytokines will activate NF-κB by degrading the IkB complex. NF-κB is translocated into the nucleus and binds to DNA and activates the transcription. The role in carcinogenesis depends on the activation of NF-κB and production of related cytokines such as IL-1, IL-2, IL-6, IL-10 and TNF-α [52]. TNF-α and interleukins can be regulated by the NF-κB transcription factor. This will suppress apoptosis, induced cellular proliferation, invasion, metastasis, chemoresistance, and inflammation. It is believed that TLRs are involved in tumor growth and development. However, activation TLRs can have anti- or pro-tumoral effects in tumor growth or metastasis in different cancer cells (Table 1) [12].

Tumorigenesis is a multistep process that can be activated by various factors such as environmental carcinogens, inflammatory mediators, and tumor promoters. The role of TLR4 in tumor progression has been described in many studies. The activation of TLR4 increases IL-8 and IL-6 production in breast cancer [54]. In addition, TLR4 activation increases expression of VEGF and TGF-β1 in prostate cancer cells, which promote tumor development [55]. In colon cancer, the TLR4 stimulation induce nitric oxide and IL-6 production [56]. Moreover, studies also shown that TLR4 overexpression is associated with poor outcome in colon and breast pancreatic cancer [54,56]. The research indicated that the MyD88 pathway of TLR-4 promoted carcinogenesis. However, studies also shown that TLR4 displayed anti-tumor activity in skin cancer [57]. The role of TLR4 should be further examined in the different tumor types. All the above suggest that the release of various cytokines, inflammatory mediators, and chemokines active TLR4 and it could contribute to cancer formation.

## 4. Bioactive Phytochemicals Targeting TLR4 and Oxidative Stress

It is well known that plants widely exert various biological properties including anti-inflammatory and anti-cancer effects. The chemical structure of TLR4 antagonist from plants is not similar to LPS. The phytochemicals that inhibit TLR4 activation are from the results demonstrating that phytochemicals decreased LPS-induced expression of cyclooxygenase-2, NF-κB and cytokine production. The pharmacological activity of phytochemicals against inflammation by targeting the TLR4 signaling pathway in many studies have been previously described [58,59,60,61,62].

Free radicals, such as hydroxyl, superoxide alkoxyl, and peroxyl (RO2^•^) radicals, are molecules with unpaired electrons in the outer orbit and are generally unstable and reactive. Excessive levels of ROS directly cause cell membrane, DNA, and protein damage. It will result in inflammation or mutation. The human body has defense mechanisms against ROS-induced damage using enzymes such as catalase and glutathione peroxidase. Excessive levels of ROS can result in adverse effects such as artherosclerosis, inflammation and cancer. A lot of studies have shown that dietary plants have natural antioxidants and intake of these antioxidants can remove free radicals and prevent some diseases. Growing in vitro and in vivo studies have shown that chemopreventive agents can enhance or regulate different signaling pathways such as PI3/AKT, NF-κB, COX-2, apoptotic, cell cycle for the treatment or prevention of different cancer cells.

### 4.1. Zingiberaceae Family

*Curcuma longa* (turmeric) and *Zingiber officinale* (ginger) belong to Zingiberaceae family and were used for different purposes for over a thousand years [63,64]. We will introduce the active components of turmeric and ginger.

#### 4.1.1. Curcumin

Turmeric is used in traditional medicine especially in China and India. The polyphenol curcumin is from rhizomes of *Curcuma longa* and widely used as a food flavoring. Curcumin has many pharmacological properties including anti-oxidant, anti-inflammatory, anti-cancer, antiproliferative, neuroprotective, hepatoprotective, immunomodulatory and chemopreventive effects [65,66]. Curcumin is a hydrophobic molecule and practically insoluble in water. The half-life of curcumin is 10 min in phosphate buffer at physiological pH 7.4 [67]. The curcumin of plasma level is very low even at high dose intake. It has been shown to exhibit anti-inflammatory effects by down-regulating cytokines, such as TNF-α, IL-1, IL-6, IL-8, IL-12, MCP-1, IL-1β, and transcription factors. Studies have shown that curcumin can compete with LPS for TLR4 and inhibition in MyD88-dependent pathway [68,69,70]. Previous studies have shown that curcumin can inhibit LPS-induced inflammation in vascular smooth muscle cells in TLR4-MAPK/NF-κB pathways by blocking ROS production [69]. These studies have shown that curcumin-decreased cytokine production includes TNF-α, IL-6 and IL-1β. Curcumin improves TNBS-induced colitis in rats via the TLR4/NF-κB signaling pathway [71]. Curcumin also possesses neuroprotection activity. Curcumin administration can reduce activation of microglia/macrophages and neuronal apoptosis through a mechanism involving the TLR4/MyD88/NF-κB signaling in experimental traumatic brain injury [70]. Recently, a study has shown that curcumin inhibits DAMP molecule HSP70 and TLR4 signaling in liver cancer cells [72].

#### 4.1.2. Ginger

Ginger is popularly used as spice and traditional medicine in China and Asian countries. It was used in traditional medicine in the treatment of various diseases such as nausea, vomiting, abdominal pain and muscle discomfort. Gingerols, shogaols, gingediols, zingerone, dehydrozingerone, gingerinone, and diarylheptanoids are extracted from rhizomes of ginger. These active constituents of ginger have many pharmacological properties including anti-inflammatory, analgesic, anti-oxidation, and anti-cancer effects [73,74,75].

6-Gingerol is identified as the main active constituent of fresh ginger. In a study, 6-gingerol decreased IL-1β-induced inflammation and oxidative stress in ROS/NF-κB/COX-2 signaling pathway in human HuH7 hepatocyte cells [76]. Additionally, orally administered 6-gingerol reduced the levels of TNF-α and expression of NF-κB and VEGF in the retinal tissue of diabetic rats [77]. Recently, a study has shown that 6-gingerol decreased production of NF-κB, TNF-α, expression of TLR4, intercellular adhesion molecule (ICAM), vascular cell adhesion molecule (VCAM), iNOS and COX-2 in liver fibrosis rats.

Gingerols undergo dehydration to form shogaols. Shogaols are a major component of dried ginger powder. 6-Shogaol has anti-cancer, antiproliferative, and anti-inflammatory effects [78]. 6-Shogaol inhibited LPS-induced TNF-α, IL-1β, IL-6, and PGE2 production and NF-κB activation in BV2 microglia cells [79]. Additionally, a study reported that 6-shogaol could inhibit iNOS, COX-2 gene expression and NF-κB activation in murine macrophages [80]. 6-Shogaol also inhibits the LPS-induced TLR4 dimerization, leading to the inhibition of NF-κB activation and COX-2 expression [81]. 6-Shogaol not only inhibits MyD88-dependent but also inhibits TRIF-dependent signal pathways in LPS-induced macrophages [82]. A recent study indicated 6-shogaol sensitized gemcitabine treatment and down-regulate NF-κB activity with its target genes COX-2, cyclinD1, survivin, cellular inhibitor of apoptosis protein-1 (cIAP-1), and X-linked inhibitor of apoptosis protein (XIAP) expression in pancreatic cancer cells. Taking these studies together, 6-shogaol can inhibit the growth of human pancreatic cells by suppressing of TLR4/NF-κB-mediated inflammatory pathways and modulate TLR-mediated inflammatory responses. 6-Shogaol might be used for the treatment of chronic inflammatory diseases or cancers.

1-Dehydro-10-gingerdione is also from ginger exerts. 1-Dehydro-10-gingerdione is more potent than 6-shogaol at inhibiting the production of NO and NF-κB activation in LPS-activated macrophages [83,84]. A study has reported that 1-dehydro-10-gingerdione binds to MD2 and downregulates NF-κB, TNF-α, IL-1β, IRF3, IFN-β and IP-10 in LPS-activated macrophages [85].

### 4.2. EGCG

Tea has been popular in China and Asia for nearly five thousand years. Green tea is prepared by steaming freshly harvested leaves. There are many bioactive compounds extracted from *Camellia sinensis* including polyphenolic substances such as epicatechin-3-gallate (ECG), epigallocatechin (EGC), epigallocatechin-3-gallate (EGCG), and epicatechin (EC). Polyphenols are one of the biggest class of phytochemicals. Polyphenols can divide into two groups: flavonoids and non-flavonoids. The polyphenols chemical structure shares basic polyphenolic structure with a single phenol ring, including phenolic acids and phenolic alcohols. These active bioactive compounds have many pharmacological effects including anti-oxidant, anti-inflammatory and anti-cancer properties [86,87]. It has been previously reported that EGCG has anti-invasive effects and inhibits activation of NF-κB and AP-1 in ECV304 human endothelial cells [88]. Furthermore, EGCG can decrease inflammatory gene expression including COX, NO synthase, and TNF-α [89]. A study also has shown that EGCG inhibits MyD88-dependent signaling pathways and TIR domain-containing adaptor inducing IFN-β (TRIF)-dependent signaling pathways of TLRs in RAW264.7 cells [90]. 67-kDa laminin receptor (67LR) is a nonintegrin cell-surface receptor. The role of 67LR is cell adhesion to the basement membrane and the metastasis of cancer cells. A study has shown that 67-kDa laminin receptor (67LR) as a cell-surface EGCG receptor and mediates the anti-cancer effects. EGCG can upregulate Tollip (Toll-interacting protein) and down-regulate of TLR4 expressions via 67LR may be effective in the anti-cancer activity [91,92].

### 4.3. Luteolin

Luteolin is a flavonoid compound found in many herbal extracts. Luteolin has anti-inflammatory, anti-metastasis, ani-oxidation, induction of apoptosis properties. Luteolin inhibited NF-κB, TNF-α, and ICAM-1 expression induced by LPS [93]. Furthermore, a study also has shown that luteolin inhibited TBK1-kinase activity in the MyD88-independent signaling pathway [94].

### 4.4. Quercetin

Quercetin (3,3′,4′,5,7-tetrasulphate) is also a flavonoid compound from vegetables and fruits. Quercetin has anti-inflammatory, anti-cancer and anti-oxidation activity. Studies have reported that quercetin can inhibit TLR4 mediated LPS-induced gene and protein expressions of inflammatory mediators and cytokines including NF-κB, COX-2, NO, PGE2, iNOS, TNF-α, IL-1β, and IL-6 [95]. NF-κB can directly regulate the transcription of pro-inflammatory cytokines including vascular cell adhesion molecule-1 (VCAM-1) and intercellular adhesion molecule-1 (ICAM-1). Quercetin can suppress the overexpression of adhesion molecules and chemokine such as VCAM-1, ICAM-1, MCP-1, expression of TLR2 and TLR4 and nuclear translocation of NF-κB p65 subunit in atherosclerosis rats [96]. A study has shown quercetin inhibited LPS-induced inflammation via inhibition of Src- and Syk-mediated PI3K tyrosine phosphorylation and TLR4/MyD88/PI3K signaling pathways [97].

### 4.5. Resveratrol

Resveratrol (3,5,4′-trihydroxy-trans-stilbene) is a natural stilbene found in peanuts, grapes, blueberries, rhubarb and wine. Resveratrol has many pharmacological properties including anti-inflammatory, chemopreventive, anti-cancer, cardioprotective, neuroprotective and hepatoprotective properties [98]. Resveratrol induced apoptosis through p53 dependent pathway [99]. Resveratrol decreased the expression of inflammatory markers as COX2, iNOS and NF-κB activation [100]. Several studies have shown resveratrol regulate the expression of TLR4. Therefore, resveratrol can be used for TLR-mediated inflammatory responses and chronic diseases associated with TLR activation. A study indicated that resveratrol decreased NF-κB activation and COX-2 expression in LPS-induced RAW264.7 and inhibited TBK1 and RIP1 in TRIF complex in MyD88-independent signaling pathways [101]. Resveratrol also decreases LPS-induced pro-oxidant effect in AR42J cells via a Myd88-dependent signaling pathway [102]. Together, these results demonstrate that resveratrol displays anti-inflammatory effects in Myd88-dependent signaling pathway or Myd88-independent signaling pathway in different experimental models. Additional study shown that resveratrol can reduce LPS-induced inflammatory responses and the down-regulation of NF-κB activity in human colon cancer cells [103]. Recently, a study further showed that resveratrol has anti-inflammatory effects by attenuating TLR4-TRAF6, MAP kinase and AKT pathways in LPS-induced macrophages [104].

### 4.6. Caffeic acid Phenethyl Ester

Caffeic acid phenethyl ester (2-phenylethyl (2E)-3-(3,4-dihydroxyphenyl) acrylate is natural compound and obtained from propolis. Studies have reported that caffeic acid phenethyl ester has anti-inflammation, anti-infections, anti-oxidative stress and anti-cancer properties [105]. Caffeic acid phenethyl ester decrease IL-12 production and NF-κB activation induced by LPS in monocyte-derived dendritic cells [106]. Additional study also shown that caffeic acid phenethyl ester prevent TLR4 activation by interfering with interaction between TLR4/MD2 complex [107]. Caffeic acid phenethyl ester can down-regulate TLR4, MyD88, IRAK4, TRIF and NF-κB p65 expression, induce cell apoptosis, and induce autophagy during the process in LPS-induced breast cancer cells [108]. Caffeic acid phenethyl ester also inhibits LPS-induced IL-6, IL-8, iNOS, and COX-2, TLR4, MyD88, NF-κB activation, PI3K and AKT phosphorylation in gingival fibroblasts cells [109].

### 4.7. Xanthohumol

Xanthohumol (E)-1-[2,4-Dihydroxy-6-methoxy-3-(3-methylbut-2-enyl)phenyl]-3-(4-hydroxyphenyl)prop-2-en-1-one) is the chalcone in the hop plant (*Humulus lupulus*). Xanthohumol is reported to elicit anti-oxidation, anti-inflammation and anti-cancer effects. Moreover, xanthohumol inhibits expression of iNOS, NO and IFN-γ production in macrophages [110,111]. Among these studies, the research found that xanthohumol has anti-inflammatory effects and inhibits TLR4 activation by binding to MD-2 (TLR4/MD-2 complex). Xanthohumol could be used in the treatment of inflammatory diseases in the future [111,112].

### 4.8. Genistein

Genistein (4,5,7-Trihydroxyisoflavone and 5,7-Dihydroxy-3-(4-hydroxyphenyl) chromen-4-one) is isolated from the dyer’s broom of *Genista tinctorial* and it is an isoflavone polyphenol. Genistein also possess many pharmacological properties including anti-inflammation. Previous in vitro and in vivo studies have revealed a down-regulation in the production of IL-6, TNF-α and NF-κB activation [113,114]. Furthermore, a study indicated genistein reduced the production of NO, PGE2, IL-1, TNF-α, TLR4 and MyD88 expression in LPS-induced BV2 microglia [115]. Genistein also strongly enhances the LPS-induced properties of MAPK transduction cascades and inhibits TLR pathway by decreasing the expression of IFN-β, IL-10, IL-1α, IL-1β, IL-6, TNF-α, CSF-2 (colony stimulating factor 2), CSF-3, CCL2 (Chemokine ligand 2) and CXCL10 (C-X-C motif chemokine ligand 10), transcription factor NF-κB, and COX-2 in macrophages [116]. Genistein also has anti-inflammatory properties in human studies. A study demonstrated daily oral genistein has beneficial effects on TNF-α levels in obese postmenopausal women after 6 months of treatment [117].

### 4.9. Berberine

*Rhizoma coptidis* is well used as an herb medicine in China. The main component of *Rhizoma coptidis* is, berberine, an alkaloid. Berberine possesses multiple properties, including neuroprotection, anti-inflammation, anti-oxidation, anti-cancer and anti-diabetes [118,119,120]. Berberine reduces the levels of IL-1β, TNF-α, iNOS, ICAM-1, IL-6, and activation of NF-κB in vivo studies [121,122]. Several studies have shown that berberine reduce inflammation though TLR4 signal pathway. Berberine inhibited Src activation and TLR-mediated cell motility in LPS-induced macrophages [123]. Additional study shown that berberine inhibited TNF-α, IL-6, TLR 2, TLR 4, and TLR 9 expression in early phase sepsis of rat [124].

### 4.10. Sulforaphane

Sulforaphane [1-isothiocyanato-4-(methylsulfinyl)butane] is extracted from cruciferous vegetables. Sulforaphane has anti-inflammatory properties by preventing IL-1R-associated kinase-1 degradation, activation of NF-κB and IFN regulatory factor 3, and COX-2 expression [125]. A study has been reported that sulforaphane acts as an anti-inflammatory molecule by suppressing TLR4 oligomerization [125]. Sulforaphane also inhibited the TLR4/MyD88 pathway and reduced the TNF-α and IL-6 levels [126]. Hypoxia-inducible factor-1 (HIF-1) can upregulate the TLR4 expression. A study has demonstrated that sulforaphane suppress hypoxia- and CoCl_2_-induced upregulation of TLR4 expression by inhibiting PI3K/AKT and HIF-1α activation [127]. Sulforaphane might provide a therapeutic target for chronic diseases related to hypoxic stress.

## 5. Prospective and Conclusions

Toll-like receptors play an important role in innate immunity. TLRs may serve as a double-edged sword in prompting cancer tumorigenesis, tumor growth, inducing apoptosis, or inhibiting tumor progression in different kinds of cancer cells or resistance to chemotherapy. Much evidence has shown that the activation of TLR4 results in inflammation and carcinogenesis. Recent studies have shown that the anti-inflammatory effects of bioactive compounds may be due to inhibition of the TLR4 pathway through MyD88- and TRIF-dependent signaling pathways. In this review article, we summarize the pharmacological properties of curcumin, 6-gingerol, 6-shogaol, 1-dehydro-10-gingerdione, EGCG, luteolin, quercetin, resveratrol, caffeic acid phenethyl ester, xanthohumol, genistein, berberine, and sulforaphane in interfere with TLR4 (Table 2). These compounds reduce the activation of the TLR4 signal pathway in a MyD88-dependent or TRIF-dependent manner. Phytochemicals interacting with TLR4 could be alternatively considered for the development of new chemoprevention because they have multiple cellular target and pharmacological properties. As we previously described, TLR4 can have pro- or anti- tumor effects in different tumor growth, progression, invasion and metastatic processes. Many pharmaceutical companies are developing TLR4 antagonists or agonists for the treatment of cancers and inflammatory diseases. Most design strategies of TLR4 antagonists use the native ligand as inspiration. Eritoran is a TLR4 antagonist and its chemical structure is similar to LPS [128]. In the future, different TLR agonists and antagonists should be low molecules, improving drug absorption, and more effectively inhibiting or stimulating TLR activity in the human body for targeting various inflammatory conditions including chemoprevention.

## Figures and Tables

**Table 1:** Pro- and anti-tumoral effects of TLR4 in cancer cells.

Effects	Tumor Type
Anti-tumor	Skin cancer, mammary cancer, lung cancer
Pro-tumor	Breast cancer, lung cancer, head and neck cancer, prostate cancer

**Table 2:** Chemical structure and molecular targets by phytochemicals.

Compound	Chemical Structure	Molecular Targets
Curcumin	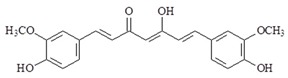	↓TNF-α, ↓IL-1, ↓IL-6, ↓IL-8, ↓IL-12, ↓MCP-1, ↓IL-1β, ↓TLR4 ↓MAPK ↓NF-κB, ↓HSP70
6-gingerol	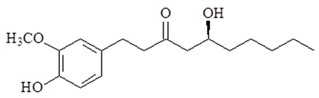	↓NF-κB, ↓COX-2, ↓TNF-α, ↓TLR4, ↓ICAM, ↓VCAM, ↓iNOS, ↓VEGF
6-Shogaol	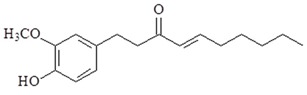	↓TNF-α, ↓IL-1β, ↓IL-6, ↓PGE2, ↓iNOS, ↓COX-2, ↓NF-κB, ↓cyclinD1, ↓survivin, ↓cIAP-1, ↓XIAP, ↓TLR4
1-Dehydro-10-gingerdione	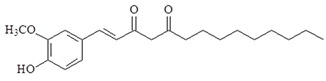	↓NO, ↓NF-κB, ↓TNF-α, ↓IL-1β, ↓IRF3, ↓IFN-β, ↓IP-10, ↓MD2
EGCG	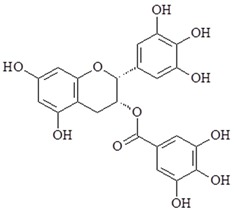	↓NF-κB, ↓AP-1, ↓COX-2, ↓NO synthase, ↓TNF-α, ↓TLR4, ↑Tollip
Luteolin	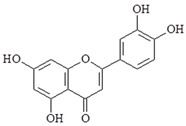	↓NF-κB, ↓TNF-α, ↓ICAM-1, ↓TBK1
Quercetin	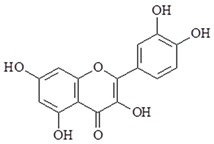	↓NF-κB, ↓COX-2, ↓NO, ↓PGE2, ↓iNOS, ↓TNF-α, ↓IL-1β, ↓IL-6, ↓VCAM-1, ↓ICAM-1, ↓MCP-1, ↓TLR2, ↓TLR4, ↓PI3K
Resveratrol	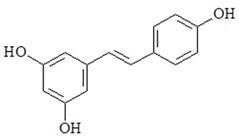	↓COX-2, ↓iNOS, ↓NF-κB, ↓TBK1, ↓RIP1, ↓TRAF6, ↓MAPK, ↓AKT, ↓TLR4
Caffeic acid phenethyl ester	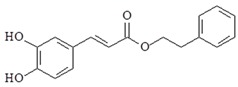	↓IL-12, ↓TLR4, ↓MyD88, ↓IRAK4, ↓TRIF, ↓NF-κB, ↓IL-6, ↓IL-8, ↓iNOS, ↓COX-2, ↓PI3K, ↓AKT
Xanthohumol	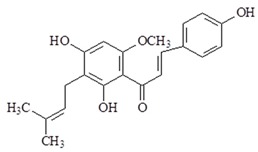	↓iNOS, ↓NO, ↓IFN-γ, ↓TLR4, ↓MD-2
Genistein	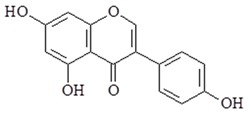	↓IL-6, ↓TNF-α, ↓NF-κB, ↓NO, ↓PGE2, ↓TNF-α, ↓TLR4, ↓IFN-β, ↓IL-10, ↓IL-1α, ↓IL-1β, ↓TNF-α, ↓CSF-2, ↓CSF-3, ↓CCL2, ↓CXCL10
Berberine	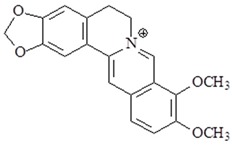	↓IL-1β, ↓TNF-α, ↓iNOS, ↓ICAM-1, ↓IL-6, ↓NF-κB, ↓TLR 2, ↓TLR 4, ↓TLR 9
Sulforaphane	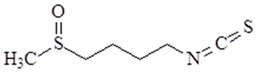	↓NF-κB, ↓TNF-α, ↓IL-6, ↓AKT, ↓HIF-1α, ↓TLR4

TNF-α = tumor necrosis factor-α; MCP-1= Monocyte chemotactic protein-1; TLR = Toll-like receptor ; NF-κB = nuclear factor kappa B; HS70 = heat shock proteins 70; AP-1 = activator protein 1; COX-2 = cyclooxygenase-2; MAPK = mitogen-activated protein kinase; IL = interleukin; iNOS = inducible nitric oxide synthase; VEGF = vascular endothelial growth factor; ICAM = intercellular cell adhesion molecules; VCAM = vascular cell adhesion molecule; PGE2 = Prostaglandin E2; cIAP-1 = cellular inhibitor of apoptosis protein-1; XIAP = X-linked inhibitor of apoptosis protein; IRF3 = Interferon regulatory factor 3; IFN-β = interferon-β; IP-10 interferon-inducible protein-10; MD2 = Myeloid differentiation protein 2; TBK1 = TANK-binding kinase 1; RIP1: Receptor-interacting serine/threonine-protein kinase 1; TRAF6 = TNF receptor-associated factor 6; AKT = Protein kinase B (PKB); MyD88 = myeloid differentiation factor 88; TRIF: TIR-domain-containing adapter-inducing interferon-β; PI3K = Phosphatidylinositol-4,5-bisphosphate 3-kinase; CSF-2 = Colony Stimulating Facto-2; CSF-3 = Colony Stimulating Facto-3; CCL2 = Chemokine ligand 2; CXCL10 = C-X-C motif chemokine ligand 10; HIF-1α = hypoxia-inducible factor-1α.↓= decrease;↑= increase.
